# A network approach to define the predictive role of immune profile on tumor response and toxicity of anti PD-1 single agent immunotherapy in patients with solid tumors

**DOI:** 10.3389/fimmu.2023.1199089

**Published:** 2023-07-07

**Authors:** Silvia Mezi, Giulia Pomati, Giulia Fiscon, Sasan Amirhassankhani, Ilaria Grazia Zizzari, Chiara Napoletano, Aurelia Rughetti, Ernesto Rossi, Giovanni Schinzari, Giampaolo Tortora, Gaetano Lanzetta, Giulia D’Amati, Marianna Nuti, Daniele Santini, Andrea Botticelli

**Affiliations:** ^1^ Department of Radiological, Oncological and Pathological Science, Sapienza University of Rome, Rome, Italy; ^2^ Department of Molecular Medicine, Sapienza University of Rome, Rome, Italy; ^3^ Department of Computer, Control, and Management Engineering “Antonio Ruberti”, “Sapienza” University of Rome, Rome, Italy; ^4^ Department of Urology, S. Orsola-Malpighi Hospital University of Bologna, Bologna, Italy; ^5^ Laboratory of Tumor Immunology and Cell Therapy, Department of Experimental Medicine, Policlinico Umberto I, University of Rome “Sapienza”, Rome, Italy; ^6^ Medical Oncology, Fondazione Policlinico Universitario Agostino Gemelli Istituti di Ricovero e Cura a Carattere Scientifico (IRCCS), Rome, Italy; ^7^ Medical Oncology, Universitá Cattolica del Sacro Cuore, Rome, Italy; ^8^ Clinical Oncology Unit, Istituto Neurotraumatologico Italiano (I.N.I.) Grottaferrata, via di S.Anna snc, Grottaferrata, Italy; ^9^ Department of Medico-Surgical Sciences and Biotechnology, Polo Pontino, Sapienza University of Rome, Rome, Italy

**Keywords:** soluble immune profile, immune-related toxicity, cytokine, chemokine, soluble adhesion molecules, soluble immune checkpoints, network analysis

## Abstract

**Background:**

The immune profile of each patient could be considered as a portrait of the fitness of his/her own immune system. The predictive role of the immune profile in immune-related toxicities (irAEs) development and tumour response to treatment was investigated.

**Methods:**

A prospective, multicenter study evaluating, through a multiplex assay, the soluble immune profile at the baseline of 53 patients with advanced cancer, treated with immunotherapy as single agent was performed. Four connectivity heat maps and networks were obtained by calculating the Spearman correlation coefficients for each group: responder patients who developed cumulative toxicity (R-T), responders who did not develop cumulative toxicity (R-NT), non-responders who developed cumulative toxicity (NR-T), non-responders who did not develop cumulative toxicity (NR-NT).

**Results:**

A statistically significant up-regulation of IL-17A, sCTLA4, sCD80, I-CAM-1, sP-Selectin and sEselectin in NR-T was detected. A clear loss of connectivity of most of the soluble immune checkpoints and cytokines characterized the immune profile of patients with toxicity, while an inversion of the correlation for ICAM-1 and sP-selectin was observed in NR-T. Four connectivity networks were built for each group. The highest number of connections characterized the NR-T.

**Conclusions:**

A connectivity network of immune dysregulation was defined for each subgroup of patients, regardless of tumor type. In patients with the worst prognosis (NR-T) the peculiar connectivity model could facilitate their early and timely identification, as well as the design of a personalized treatment approach to improve outcomes or prevent irAEs.

## 1 Introduction

The immune system is a complex entity regulated by several circulating molecules which shape and modify the tumour microenvironment in either a pro or anti-tumour direction (1). The ability of the immune network to control cancer growth can induce a continuous molecular and phenotypic remodelling of cancer cells and the tumour micro-environment, resulting in the survival of cancer cells even in an immunocompetent host. Immune checkpoint inhibitors (ICIs), which target the CTLA-4 and PD-1/PD-L1 axis and are able to remove tumour cell-induced inhibition by unlocking the anergic state of T lymphocytes, have been a real breakthrough in cancer treatment (2, 3). The development of PD-1 inhibitors such as nivolumab and pembrolizumab and of anti PD-L1 drugs such as atezolizumab and durvalumab, approved to date, has made remarkable long-term results possible, both in the first and second-line settings as well as in the adjuvant and neo-adjuvant ones (4–16). These drugs can be employed as monotherapy or in combination with other agents in various oncological diseases, including non-small cell lung cancer (NSCLC), renal cell carcinoma (RCC), and recurrent/metastatic head and neck cancer (R/M-HNSCC). In addition, immunotherapy with ICs is also currently under investigation in uveal melanoma (UM) (8–10). Although immunotherapy has deeply changed the treatment landscape and prognosis of these tumours, only a small percentage of patients achieve long-term benefit in terms of overall survival (OS).

In addition, ICIs have peculiar immune-related adverse events (irAEs) (17, 18). IrAEs may potentially affect any organ as immune cells, bypassing self-tolerance mechanisms, could act against healthy tissues (19). There are no certain explanations as to why some individuals have a greater tendency to develop irAEs than others. Patients with pre-existing autoimmune diseases have a higher risk of irAEs. Genomic profile and microbiota composition may also play an important role in the risk of irAEs and treatment activity (20). Moreover, the concomitant use of drugs such as antiarrhythmics, antibiotics, anticonvulsants or antipsychotics and opioids may play an important role in the development of severe irAE and tumor resistance (21–24). The most common irAEs involve the skin, endocrine glands, gastrointestinal system and liver (25). Compared to chemotherapy, irAEs are usually mild to moderate in severity, reversible and can be treated promptly with appropriate immunosuppressive agents, whereas severe and fatal irAEs are rare. Nevertheless, some of them may be associated with life-threatening decline in organ function, quality of life (QoL) and temporary or permanent discontinuation of immunotherapy (26, 27). Moreover, specific irAEs seem to be related to a particular type of cancer. For instance, patients with NSCLC tend to develop irAEs earlier, with a higher incidence of interstitial pneumonia than those with melanoma (28, 29).

Currently available data suggest that soluble molecules play a central role both in determining response to immunotherapy and in the occurrence of irAEs. Understanding the patient immune status may lead to the identification of those who will have the worst clinical outcomes requiring alternative and targeted therapeutic strategies. Therefore, the identification of predictive validated biomarkers of immunotherapy-related toxicities and treatment resistance represents an urgent unmet need.

The patient soluble immune profile could become a reliable predictive factor, as soluble molecules are involved in the immune-fitness as well as in the dysfunctional activity of the immune system and constitute a repeatable and non-invasive method of monitoring the patient immune profile (30–34).

The aim of this study was to define whether the patient immune profile could show a predictive role in the response to therapy and irAEs development in patients with metastatic solid cancers treated with single agent immunotherapy. Predicting the occurrence of both resistance to therapy and irAEs would give the opportunity to modulate and tailor the therapeutic strategy based on the immune characteristics of each individual patient, thus preventing ineffective treatments which could compromise QoL by causing failure of response to therapy and/or life-threatening irAEs.

## 2 Materials and methods

### 2.1 Patients enrollment and sample collection

From April 2017 to May 2021, 53 patients with metastatic NSCLC, UM, R/M-HNSCC and RCC, who received immunotherapy, with either nivolumab or pembrolizumab, were enrolled in this prospective, multicentre study. ICI treatment was administered intravenously as first or second line setting, according to approved schedule, until either disease progression, development of unacceptable toxicity or patient refusal occurred. Patient characteristics, including Eastern Cooperative Oncology Group (ECOG) Performance Status (PS), age, gender, histology and previous treatments, were recorded. Patients were clinically staged with contrast enhanced computed tomography (CT) scan and, if clinically indicated, magnetic resonance imaging (MRI) and CT/PET at baseline (T0) and every 3 months. Patients aged 18 years or older with advanced/metastatic solid tumours, fit for immunotherapy with adequate bone marrow, liver, and renal function, and ECOG PS ≤ 1, were included. All patients provided an informed consent to be included in the study and for blood samples to be collected. Patients who received anti-neoplastic immunotherapy for other previous or concomitant pathologies, with PS≥2, with uncontrolled autoimmune or infectious diseases or not compliant with protocol requirements were excluded. Patient blood samples were collected at T0, before starting anti-PD1 treatment, into BD Vacutainer Plus Plastic Serum tubes (Becton Dickinson, NJ, USA) and processed within 1 hour after blood sampling. Afterwards, tubes were centrifuged at 1800 rpm for 10 minutes. Patient serum was collected and stored at -80°C until use. Patients characteristic are described in **Table 1**.

**Table 1:** Baseline clinical and pathological characteristics in overall patients population and in each clinical group. Association between clinical/pathological characteristics and four clinical groups is reported.

CHARACTERISICS	PATIENTS (%)	NR-T	NR-NT	R-T	R-NT	*P value*
Age						
Median Range	71 (50-89)	75 (57-89)	72 (50-89)	69 (62-81)	73 (57-83)	0.85**
Gender						
Male	33 (63)	8 (73)	15 (65)	5 (71)	5 (42)	0.389
Female	20 (37)	3 (27)	8 (35)	2 (29)	7 (58)	
Cancer type						
NSCLC	14 (26.4)	2 (19)	8 (34)	2 (29)	2 (17)	0.00001*
UM	18 (34%)		12 (52)		6 (50)	
R/M HNSCC	13 (24.5)	9 (81)	1 (4)	1 (11)	2 (17)	
RCC	8 (15.1)		2 (9)	4 (57)	2 (17)	
Previous treatment						
No treatment	18 (34)	0	12 (52)	0	6 (50)	0.00001*
Chemotherapy	27 (51)	11 (100)	9 (39)	3 (43)	4 (33)	
Target therapy	8 (15)	0	2 (9)	4 (57)	2 (17)	
Immunotherapy						
Nivolumab	35 (66)	11 (100)	11 (48)	7 (100)	6 (50)	0.03*
Pembrolizumab	18 (34)	0	12 (52)	0	6 (50)	
Line of ICI Treatment						
First Line	18 (34)	0	12 (52)	0	6 (50)	0.003*
Second Line or more	35 (66)	11 (100)	11 (48)	7 (100)	6 (50)	

* p value ≤0.05 was considered statistically significant.

** p-value was obtained via Kruskal-Wallis test.

### 2.2 Tumor response to treatment

Best tumor response was assessed using immune-related Response Evaluation Criteria in Solid Tumors (i-RECIST) and classified as complete response (CR), partial response (PR), stable disease (SD), and progressive disease (PD). Based on the response to immunotherapy, patients were classified as non-responders, if progression occurred at the first clinical-instrumental evaluation after the start of immunotherapy, or responders, if the best response was stable disease (SD) or partial response (PR) for at least 4 months. Data were collected anonymously into a specific database. Protocol approval from Local Ethics Committee was obtained [CE 4421].

### 2.3 Toxicities

Patients were evaluated at the time of each administration of the drug through blood tests and clinical assessment. Any AEs were recorded at each cycle and classified according to the National Cancer Institute Common Terminology Criteria for AEs (version 4.0). IrAEs have been defined as either low grade (G1) or high grade (G2-G3). Cumulative toxicity was defined as the presence of at least two irAEs of any grade (35). For each patient, the treatment of irAEs was carried out through multidisciplinary discussion with endocrinologists, rheumatologists, nephrologists and dermatologists, as suggested (36–38).

### 2.4 Serological evaluation of immune-related molecules

Serum, collected at baseline (T0), was assayed to detect the concentration of 12 cytokines, 5 chemokines, 13 sICs, 3 adhesion molecules and IDO. Levels of soluble immune related molecules were dosed through a multiplex assay using the ProcartaPlex Human Inflammation Panel (20 Plex, catalog number EPX200-12185-901; sE-Selectin; GM-CSF; ICAM-1/CD54; IFN alpha; IFN gamma; IL-1 alpha; IL-1 beta; IL-4; IL-6; IL-8; IL-10; IL-12p70; IL-13; IL-17A/CTLA-8; IP-10/CXCL10; MCP-1/CCL2; MIP-1alpha/CCL3; MIP-1 beta/CCL4; sP-Selectin; TNF alpha) (eBioscence, Vienna, Austria) and the Human Immu-no-Oncology Checkpoint 14-Plex ProcartaPlex Panel 1 (catalog number EPX14A-15803-901; BTLA; GITR; HVEM; IDO; LAG-3; PD1; PD-L1; PD-L2; TIM-3; CD28; CD80; CD137; CD27; CD152) (eBioscence) according to manufacturer instruction. Samples were measured using Luminex 200 platform (BioPlex, Bio-Rad) and data, expressed in pg/ml of protein, were analyzed using Bio-Plex Manager Software.

### 2.5 Statistical analysis

Statistical analysis was performed by using R statistical software (R Foundation for Statistical Computing, Vienna, Austria; version 4.0.4; URL https://www.R-project.org). A total of 34 molecules, extracted from a cohort of 53 patients were analyzed. Patients were stratified in four clinical scenarios: 7 responder patients who developed cumulative toxicity (RT), 12 responder patients who did not develop cumulative toxicity (R-NT), 11 non-responder patients who developed cumulative toxicity (NR-T), 23 non-responder patients who did not develop cumulative toxicity (NR-NT). A connectivity profile of each of these clinical setting was built. Data were first pre-processed by applying a base-2 logarithmic transformation. Then, experimental differences of the molecules expression levels from the four patient groups were tested for statistical significance, both among all groups and between each pair of groups by using the Kruskal Wallis test and the Mann-Whitney test, respectively, at T0 (i.e., basal). P-values were adjusted for multiple comparisons by using FDR correction; an adjusted p-value of 0.05 or less was considered to be statistically significant.

Chi-square (for large-sized samples) or Fisher’s exact tests (for small-sized samples) were employed to assess the association between two categorical variables of interest, i.e., the clinical/pathological features in regards to the four clinical groups (NR-T, NR-NT,R-T,R-NT) or to the three best response groups (PD, SD, RP) (39). A p-value of 0.05 or less was considered to be statistically significant. Differences of the clinical continuous variable (i.e., age) within the four patient groups (or in the best response group) were tested for statistical significance, both among all groups (by means of Kruskal-Wallis test) and between each pair of groups (via Mann-Whitney test).

### 2.6 Connectivity analysis

In order to investigate the relationships between the therapy response and the toxicity, we analyzed the differences in terms of the connectivity exerted by the soluble molecules in responder patients and non-responder patients with and without toxicity. In particular, four connectivity matrices were built by calculating the Spearman correlation coefficients (and the corresponding p-values) among each pair of molecules for each group of analyzed patients (R-T, R-NT, NR-T, NR-NT). Thus, the four matrices were rendered as four connectivity maps where correlation values increase shifting from red to blue. P-values associated to each correlation values were adjusted for multiple comparison and an adjusted p-value of 0.05 or less was considered to be statistically significant. Then, four corresponding networks of connectivity were then constructed, in which nodes represented molecules and a link occurring between them if the absolute value of Spearman correlation between their expression levels was greater than a selected threshold (i.e., the 80th percentile of the overall distribution corresponding to 0.7) and statistically significant (adjusted p-value ≤ 0.05). All the connectivity networks along with their corresponding values of correlation and statistical p-values were detailed as lists in tables.

## 3 Results

### 3.1 Patients

Fifthy-three metastatic patients treated with anti PD-1 agent were enrolled in this study: 18 patients with UM, 8 patients with RCC, 13 with HNSCC, and 14 with NSCLC. Baseline clinical–pathological characteristics of patients are summarized in **Table 1**. All 8 patients in the RCC group had clear cell carcinoma and all 13 patients with HNSCCs had squamous histology. All patients with NSCLC were non-oncogene addicted: 11 cases were squamous cell carcinoma and 2 were adenocarcinoma. Thirty-three patients were male (63%), 20 patients were female (37%). The mean age was 71 years (50-89). All patients were treated with either nivolumab or pembrolizumab: 18 patients in a first-line setting treated with pembrolizumab and 35 patients in a second line setting, treated with nivolumab.

Clinical-pathological characteristics of patients in each clinical group (NR-T, NR-NT, R-T, R-NT) are reported in **Table 1**. Statistical significance association was found between the four clinical groups and the following clinical/pathological characteristics: cancer type (p = 0.00001), treatment line (p = 0.003), previous treatment (p = 0.00001), immunotherapy (p = 0.03). No statistical significance was found for gender (p = 0.389) and age variable (p = 0.8) in the four clinical groups.

### 3.2 Response to immunotherapy and toxicities

The outcomes of immunotherapy, in terms of best response, are shown in **Table 2**. Thirty-four patients (64.1%) experienced progressive disease as the best response, while 15 patients (28.3%) had stable disease and 4 patients (7.6%) partial response.A statistically significant association was found between best response and cancer type (p = 0.02). No significant association was found between best response and toxicity (p = 0.183), gender (p = 0.325), age (p = 0.8), treatment line (p = 0.312), and immunotherapy drug (p = 0.312).Toxicities occurred in 28 patients (52.8%). Twenty-five (47%) patients reported G1 toxicity and 16 (30.2%) patients reported G2-G3 toxicity. In addition, 18 (34%) patients developed more than one toxicity during therapy, reporting the presence of at least two irAEs (**Table 3**). Neither immune related deaths, nor any unexpected toxicity were recorded. There was no discontinuation of treatment due to adverse events, as the G3 toxicities occurred in 2 patients concomitantly with disease progression.

**Table 2:** Best response in the overall study population and in each type of primary tumor. Association between primary tumor type and best response group is reported.

PARAMETER	PATIENTS (%)	UM (%)	RCC (%)	HNSCC (%)	NSCLC (%)	*p* value
	53	18	8	13	14	
PROGRESSIVE DISEASE	34 (64.1)	12 (66.7)	2 (25)	10 (77)	10 (77.4)	0.02*
STABLE DISEASE	15 (28.3)	6 (33.3)	3 (37.5)	3 (23)		
PARTIAL RESPONSE	4 (7.6)	–	3 (37.5)	.	1 (7.2)	

* p value ≤0.05 was considered statistically significant.

**Table 3:** Patients reporting toxicities; type and grading.

CHARACTERISICS	PATIENTS (N)	(%)
**Any grade Toxicities**	28	52.8%
Toxicity G1	25	47%
Toxicity G2-G3	16	30.2%
Cumulative Toxicities	18	34%
Asthenia	20	37.7%
Skin Toxicity	14	26.4%
Endocrine toxicity	8	15%
Gastro-intestinal	8	15%
Arthritis/arthralgia	4	7.5%
Mucositis	3	5.7%
Neurological symptoms	2	3.8%
Hematologic	2	3.8%
Ophtalmic	1	1.9%

The most common toxicity was non-specific asthenia, reported in 20 patients (37.7%). The other toxicities were: skin toxicity in 14 patients (26.4%), endocrine toxicity in 8 (15%), gastrointestinal in 8 (15%), arthritis/arthralgia in 4 (7.5%), mucositis in 3 (5.7%), neuro-logical symptoms in 2 (3.8%) patients, haematologic toxicity in 2 (3.8%) patients and ophthalmic toxicity in 1 (1.9%) case.

### 3.3 Statistical analysis of circulating molecules in responder and non-responder patients with and without toxicity

**Figure 1:**
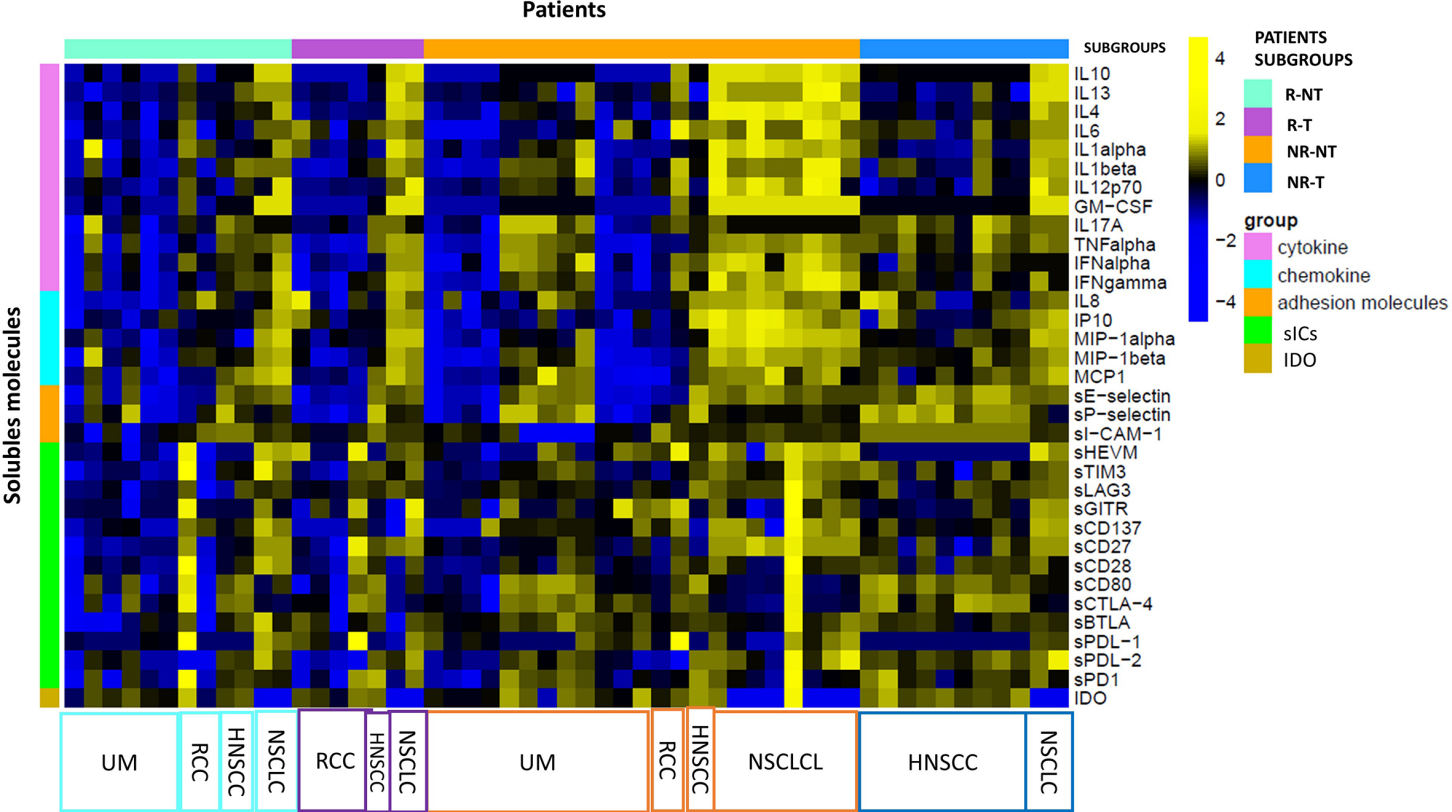
Statistical analysis at T0. Heatmap of expression levels of immune mediators (base-2 loga-rithmic scale) at T0 across 53 patients grouped by R-T (violet bars), R-NT (water blue bars), NR-NT (orange bars), NR-T (blue bars). A z-score normalization was applied and colors represent different expression levels increasing from blue to yellow. The distribution of primary tumours in each subgroup is indicated at the bottom of the heatmap.

**Figure 2:**
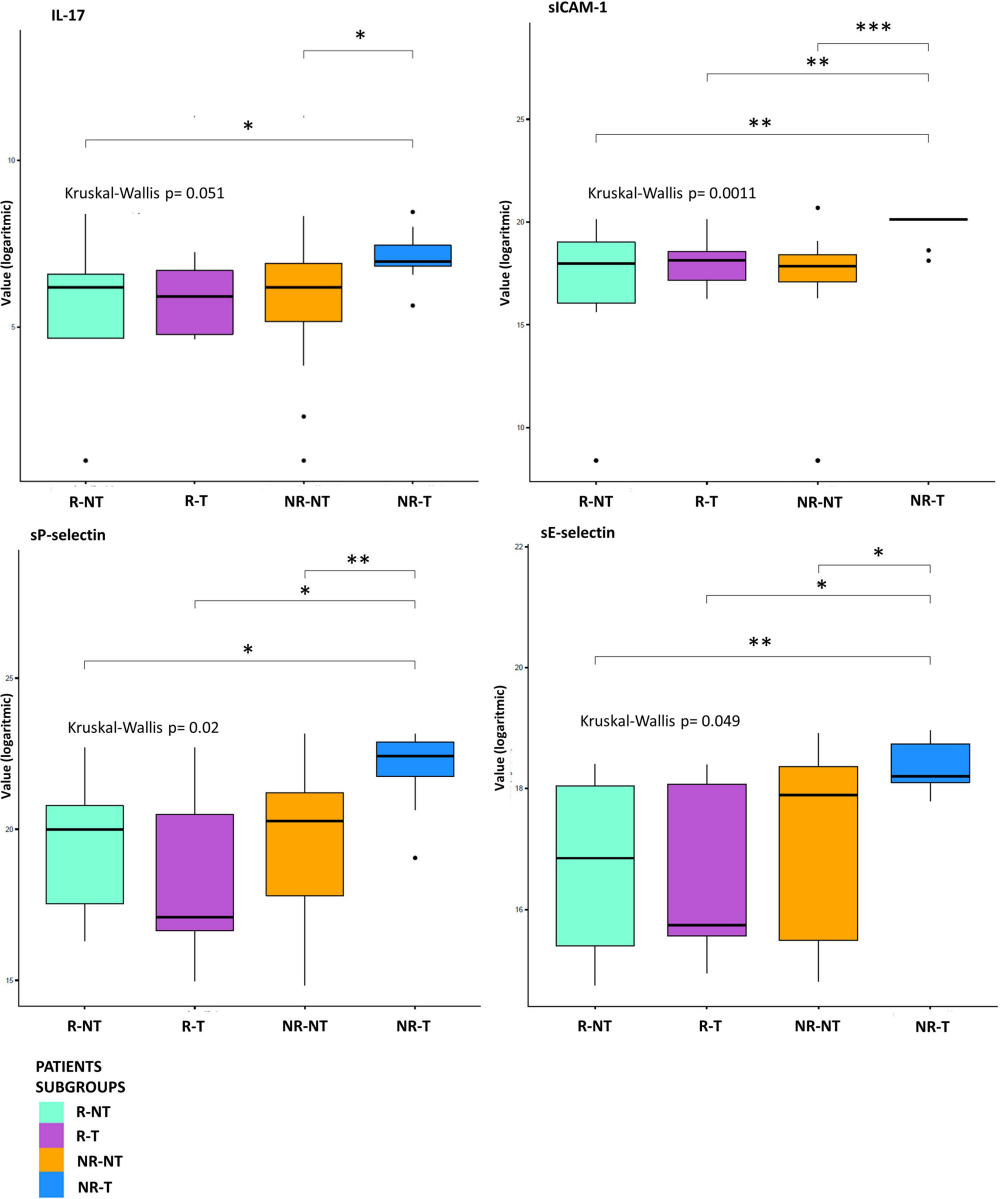
Statistical analysis at T0. Boxplot of molecules expression level (base-2 logarithmic scale) in 7 R-T (violet box), 12 R-NT (water blue box), 23 NR-NT (orange box) and 11 NR-T (blue box) at T0. Pairwise p-values (p) were obtained employing a Mann-Whitney test for unpaired samples, while overall p-value was obtained via Kruskal-Wallis test. Only molecules showing an overall statistically significant difference among all groups and a pairwise statistical difference in at least one comparison are shown. Legend: * p-value ≤ 0.05; ** p-value ≤ 0.01; *** p-value ≤ 0.001.

**Figure 3:**
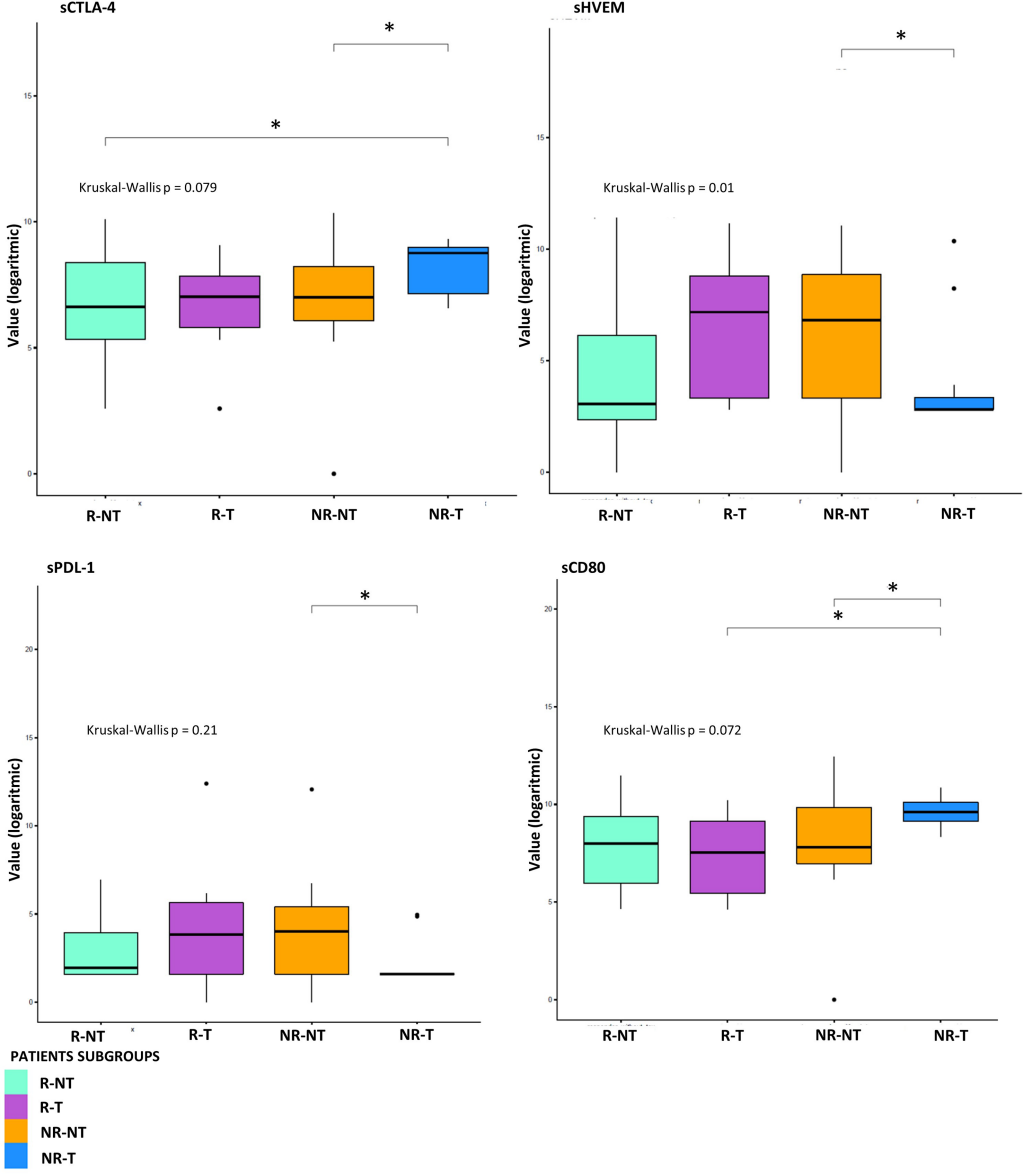
Statistical analysis at T0 for sICs. Boxplot of sICs expression level (base 2 logarithmic scale) in 7 R-T (violet box), 12 R-NT (water blue box), 23 NR-NT (orange box) and 11 NR-T (blue box) at T0. Pairwise p-values (p) were obtained emplying the Mann-Whitney test for unpaired samples, overall p-value was obtained via Kruskal-Wallis test. Legend: * p-value ≤ 0.05.

Exploratory data analysis of the 34 molecules from 53 patients grouped by therapy response with or without cumulative toxicity, was performed. Four clinical groups were analysed: 12 R-NT patients (6 UM, 2 RCC, 2 HNSCC, 2 NSCLCL), 23 NR-NT patients (12 UM, 2 RCC, 1 HNSCC, 8 NSCLC), 11 NR-T patients (9 HNSCC, 2 NSCLCL), 7 R-T patients (4 RCC, 1 HNSCC, 2 NSCLCL). There was no clear separation in terms of overall molecule expression levels between the four classes (**Figure 1**). Statistically significant differences in the four groups for the cytokine IL-17A and all of the three adhesion molecules (i.e., s-ICAM-1, sP-selectin, sE-selectin) were detected (**Figure 2**). A statistically significant up-regulation of the cytokine IL-17A and all of the adhesion molecules in NR-T group, compared to the other ones, was observed (**Figure 2**), regardless of the primary tumor type. Furthermore, some immune-checkpoints, including sHEVM, sCTL4-1 and sPDL1 showed statistically significant differences between NR-T and NR-NT groups (**Figure 3**). CTLA-4 was significantly higher in NR-T compared to the R-NT group; sCD80 was significantly higher in NR-T than in both the NR-NT and R-T groups (**Figure 3**). On the other hand, both sHVEM and sPDL1 were significantly lower in the NR-T group than in the NR-NT one.

### 3.4 Connectivity analysis between circulating molecules in responder and non-responder patients with and without toxicity

**Figure 4:**
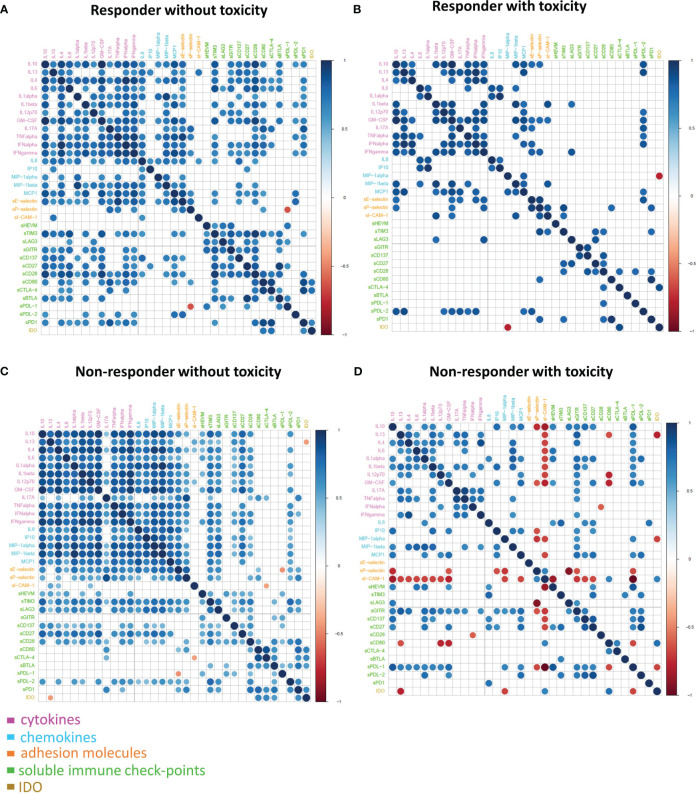
Connectivity map between molecules in R-NT **(A)**, R-T **(B)**, NR-NT **(C)**, NR-T **(D)** at T0. Statistically significant Spearman correlations (p-value ≤ 0.05) are reported. In the plot, circles are scaled and coloured according to the correlation values, increasing from red (negative correlation) to blue (positive correlation). Molecules are grouped and ordered according to the functional group reported in the legend.

To investigate the difference in the molecule connectivity patterns in terms of therapy response and toxicity,four connectivity maps were built between each pair of the molecule concentration values in R-NT and R-T (**Figures 4A, B**) as well as in NR-NT and NR-T (**Figures 4C, D**) considering all the different cancer types as a whole.

These maps showed clearly different connectivity patterns in patients either with or without toxicity. Most notably, a loss of connectivity of most of the sICs and the cytokine correlations between the groups was observed (e.g., all the connections of the pro-inflammatory cytokines IL13, IL6, IL-17A, TNFalpha, most of the connections of the other groups of chemokines including IL8, MIP-I-alpha). This could be observed comparing the connectivity map of both R-NT and NR-NT groups with their toxicity counterparts. Moreover, a change of the correlation for sICAM-1 and sP-selectin was detected in the NR-T group, where the correlation became inverse from a previously direct one (e.g., the correlation of s-Pselectin with the cytokines IL10 and GM-CSF appears positive in NR-NT, but it becomes negative in NR-T), or an otherwise absence of correlation changed to an inverse one (e.g., s-ICAM-I appears with no correlation with almost all the cytokines in both R-NT and NR-NT and R-T, while it turned on with a strong negative correlation in NR-T).

The NR-T group is distinguished from the other 3 by the occurrence of strongly negative correlations. In particular:

- between ICAM-1 and sHVEM, sPDL-1 and many other cytokines (IL-10, GM-CSF, IL13, IL4, IL6, IL1 alpha, IL1 beta and IL12p70);
- between sP-selectin and IL10, GM-CSF, IP10, MIP1alpha, LAG3, sGITR and sPDL1;
- between sCD80 and IL13, GM-CSF, IL12p70, sPDL-1;
- between IDO and IL13, MIP-1alpha and sPDL-1.

**Figure 5:**
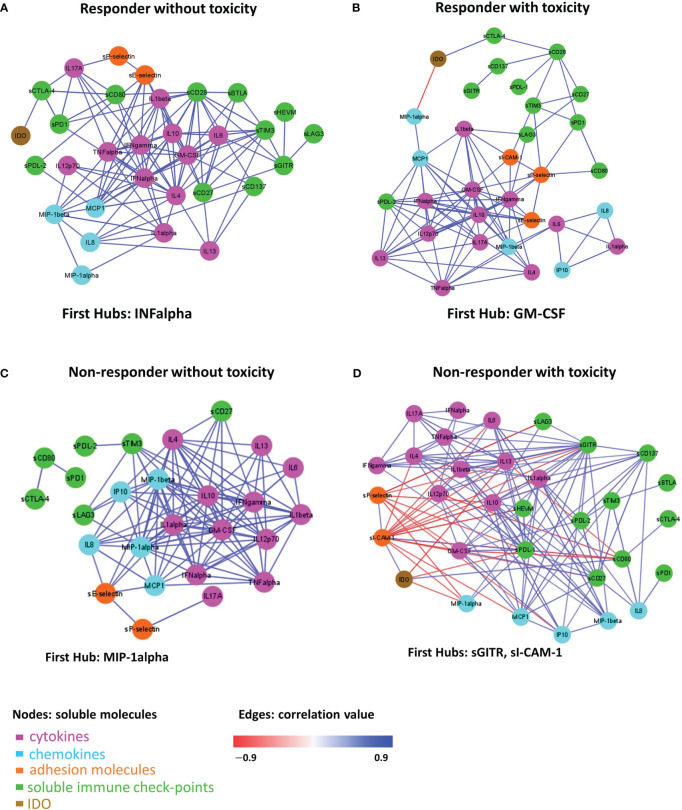
Connectivity network between molecules in R-NT **(A)**, R-T **(B)**, NR-NT **(C)**, NR-T **(D)** at T0. In each network, nodes represent molecule and a link occurs between two nodes if the absolute value of Spearman correlation between their expression levels is statistically significant (p-value ≤ 0.05) and greater than a selected threshold (i.e., the 80th percentile of the overall distribution corresponding to 0.7). Nodes are colored according to the functional groups reported in the legend; whereas edge colour indicates positive (blue) or negative (red) correlation values.

G2-G3 toxicities were present in all patients belonging to the NR-T group, suggesting that those with the most unfavorable clinical, prognostic and immune characteristics were concentrated in this small sample of patients. These differences are more evident when the connectivity maps for responder and non-responder patients with and without toxicity were rendered as four corresponding connectivity networks (**Figure 5**), where two nodes are connected if their expression profiles are statistically significant (p-value ≤ 0.05) and exceed in absolute value a selected correlation threshold (i.e., the 80th percentile of the overall distribution corresponding to 0.7).

A total of 111 statistically significant connections were identified in the R-NT group (**Figure 5A**, **Table S1**, first sheet), 95 statistically significant connections (correlations) in the R-T group (**Figure 5B**, **Table S1**, second sheet), 113 statistically significant connections in the NR-NT (**Figure 5C**, **Table S2**, first sheet) and finally 143 statistically significant connections in the NR-T group (**Figure 5D**, **Table S2**, second sheet). Interestingly, only 14 common connections among the four networks connecting 13 molecules (i.e., **Table S3**, first sheet) were found, while a substantial number of connections specifically observed for each group of patients came up (**Table S3**, second-fifth sheets): 26 connections in R-T, 38 in R-NT, 80 in NR-T, 31 NR-NT patients.

In addition, in the network of each group, molecules with a central hub role were identified: INF-alpha in the R-NT group, GM-CSF in the R-T group, MIP-1alpha in the NR-NT one and both sICAM-1 and sGITR in the NR-T patients.

## 4 Discussion

This study evidenced that the connectivity between the circulating molecules taken into account can change with a specific signature in responder and non-responder patients with or without immune-related toxicity.

The mechanisms by which tumour cells can evade the control of the immune system are manifold. Among these interrelated mechanisms the study of the circulating factors regulating the immune activity is attracting the highest interest in the current scientific research.These circulating molecules could contribute to identify useful biomarkers in the selection of patients who could benefit the most from immunotherapy (34, 40, 41). The interacting biological signals, involving soluble molecules, show remarkable capabilities, such as influence over tumor growth, lymphocyte recruitment, T-cell differentiation and involvement in the inflammation processes. Improper immune responses mediated by soluble molecules can cause autoimmune diseases or even promote cancer progression (1, 42–44). For this reason, 34 molecules were studied with synergistic and complementary activity within the immune system in order to assess the predictive role of the basal immune profile.

Biological systems form complex molecular networks, as they respond to multiple and varied inputs simultaneously (45). The investigation of these networks as a whole is essential since each molecular entity does not exert its effect on phenotype on its own, and diseases are driven by complex interactions among a variety of molecular mediators (46). To construct these networks, a quantitative approach, based on the co-expression between molecules, quantifies the relationship between two molecules (connectivity) by correlating their expression profiles. Although correlation does not imply causation, co-expressed molecules may have a shared mode of functioning in realizing a coordinated response to an external stimulus (47).

In this study a specific connectivity network was constructed according to the occurrence of cumulative toxicity, detecting a specific immune condition in patients susceptible to developing toxicity during treatment in agreement with previous available data (48). A lack of a statistically significant association between best response and toxicities was highlighted. These data suggest that the presence of toxicity does not represent a predictive factor of tumor response. This evidence is in contrast with what is reported in the available literature. In a recent meta-analysis, the onset of irAE was associated with better ICI activity and a survival benefit (49). This association is particularly significant for some types of cancer (melanoma and NSCLC) and for specific types of irAEs (endocrine and skin) (49). On the other hand, a statistically significant difference in the distribution of patients in the first or second line setting was found in the 4 clinical groups (**Table 1**). In particular, patients who develop toxicity are in second-line setting therapies.

Instead, a peculiar connectivity network in the NR-T subgroup was identified. In this group, higher levels of IL-17A and adhesion molecules (sICAM-1, sP-selectin and sE-selectine) were detected compared to other subgroups (**Figure 2**).

IL-17A plays a key role in fostering the creation of an ideal tumour microenvironment through its ability to induce the production of inflammatory mediators and mobilize MDSC cells (50). By binding to its receptor (IL-17Ra), IL-17A is able to promote oncogenesis and angiogenesis; thus it has been associated with a poor prognosis in colon rectal cancer patients (51, 52). Furthermore, IL-17A promotes the immunosuppressive activity of Treg cells, resulting in tumour progression (53). Recently, IL-17A has been associated with the failure of anti-PD-1 therapy in patients with MSS colon-rectal cancer (CRC) (54). Although growing evidence suggests that IL-17A activity may drive resistance to anti-tumour immunotherapy and contribute to therapeutic failure, it is still unclear if blocking IL-17A could improve sensitivity to ICIs (54). Moreover, IL-17A is implicated in the pathogenesis of some autoimmune diseases, including ankylosing spondylitis for which an IL-17A inhibitor (secukinumab) is approved (55). Therefore, IL-17A might play a key role as a driver of immune dysregulation in the NR-T subgroup. Similarly, the presence of high circulating levels of soluble adhesion molecules also confirms the loss of immune balance in patients of this subgroup. High levels of soluble adhesion molecules have been associated so far with sepsis, autoimmune diseases such as reumatoid arthritis and other inflammatory and vascular conditions (56–58). Moreover, sICAM-1 may have predictive clinical value in transplanted patients with acute renal allograft rejection, as it is present in serum in high concentrations prior to the occurrence of the acute event (59). Recently, it has been shown that hepatocellular carcinoma (HCC) patients with elevated serum levels of sICAM-1 had a worse OS than those with low levels of sICAM-1, who were more likely to benefit from immune checkpoint blockade therapy (60). Furthermore, in a recent study including patients with different primary tumours treated with immunotherapy, the worst prognosis cluster was characterized by elevated serum levels of cytokines/chemokines and adhesion molecules, confirming the possible predictive significance of imbalance of these molecules (42). These findings were confirmed in the connectivity map (**Figure 4**), showing that in the NR-T group there are specific inverse correlations between ICAM-1, sP-selectins and different cytokines/chemokines. In this subgroup higher levels of sCTLA-4 compared to both R-NT and NR-NT groups and higher levels of sCD80 compared to both R-T and NR-NT groups were found. On the other hand, sHVEM and sPDL-1 levels were lower in the NR-T group compared to the NR-NT one. The biological functions of the soluble forms of HVEM and PDL1 are still largely unknown, but some evidence suggests an important immunoregulatory role (44, 61). The soluble form of CTLA-4, capable of binding CD80, is implicated in the pathogenesis of several autoimmune diseases, in which sCTLA-4 is able to inhibit early T-cell activation by blocking the interaction between CD80 and the costimulatory receptor CD28. Furthermore, high levels of sCTLA-4 could compete for binding of the membrane form of CTLA-4 causing a reduction in inhibitory signaling (62). The potent immunoregulatory activities of sCTLA-4 are confirmed by the high concentrations in the sera of patients with melanoma who develop toxicity during immunotherapy (63).

Considering the organ-specific immunity, the majority of HNSCCs are included in the NR-T group. HNSCCs as known in the literature, are tumours characterized by an extremely immunosuppressive microenvironment, in which dysregulation of the immune system plays a central role in carcinogenesis and tumour progression (64).

These results are particularly significant, considering that the NR-T subgroup developed predominantly high grade toxicities. This set of patients, presenting the worst clinical condition, would require a targeted and customized management, directed towards alternative treatment choices or, preferably, the inclusion of the patient in specifically designed clinical trials or within the institutional molecular tumor board: candidates for molecular profiling should be allowed access to customized treatments based on the specific molecular alterations that may be highlighted, as an alternative to immunotherapy treatment.

The 4 connectivity maps obtained showed that in patients who develop toxicity, regardless of tumour response, there is a specific and peculiar connectivity pattern characterized by a loss of connectivity of most of the sIC and the cytokines/chemokines correlations suggesting that the immune system is in equilibrium when there is a well-organized crosstalk between the different molecules, whereas it is dysregulated and more ‘inflamed’ when molecules act chaotically losing connections with each other.

Although toxicity has the greatest impact on the connectivity pattern, it is possible to observe peculiarities in the connectivity map of NR-T patients, in which a reversal of correlation is observed for sICAM-1 and sP-selectin compared to the other 3 groups. These negative correlations involve, above all, two soluble adhesion molecules (s-Pselectin and sICAM-1 with a negative correlation with many cytokines/chemokines) and IDO (with the occurrence of a negative correlation with IL13, MIP1-alpha and sPDL1). These findings might suggest that the NR-T group is more “inflamed” than the other 3, with greater dysregulation and loss of immune fitness.

The differences in the 4 groups of patients are confirmed by the connectivity networks analysis, which shows the presence of specific connections for each subgroup, with a relatively small number (14) of connections shared by all 4 networks. In addition, each network has a distinct molecule with the role of leading player in the greatest number of interactions, defined as the “first hub”. Significantly, IFNα is the first hub in the connectivity network of R-NT, representing the group with the best prognosis, benefitting the most from immunotherapy. The importance of IFN-alpha in cancer control has been known for a long time, since it was the first drug approved in the treatment of certain solid tumours such as melanoma and kidney cancer (65). As a central immunomodulatory agent, IFN-alpha has an important immunomodulatory function and is able to mediate pro-apoptotic and anti-proliferative functions favoring cancer elimination. However, chronic activation of the IFN-alpha pathway may lead to an immunosuppressive action due to depletion of the immune activity involved in tumour escape mechanisms (66). In the NR-T group, sGITR and sICAM-1 were the first hub. As already mentioned, soluble adhesion molecules should play an important role in this subgroup of patients, given their pro-inflammatory action. On the other hand, the GITR/GITR ligand pathway induces a positive costimulatory signal on effector T cells, promoting their activation and proliferation, the inhibition of regulatory T (Treg) cells, the co-activation of NK-cells, activation of macrophages, modulation of DC function and regulation of the extravasation process (67, 68). GITR activation has been so far associated with anti-tumor activity, anti-viral activity and aggravation of autoimmune diseases (69). Elevated serum levels of the soluble form of GITRL and/or GITR have been reported in some autoimmune disorders such as Sjögren’s syndrome and Hashimoto’s thyroiditis (70, 71). Preclinical data on GITR-agonist monoclonal antibodies demonstrated *in vitro* and *in vivo* antitumor activity which enhances the CD8+ and CD4+ effector T cells and decreases tumour-infiltrating Tregs (72). However, the functions of the soluble form of GITR are not fully understood to date.

The first hub in the R-T group was GM-CSF, a cytokine able to promote the differentiation of myeloid cells, with immunostimulatory effects inducing antitumor immunity. Furthermore, GM-CSF is capable of inducing the differentiation of DCs, which are responsible for the presentation of tumor antigens for the priming of cytotoxic T lymphocytes (73).

In the NR-NT group MIP1-alpha was the first hub, a chemokine capable of both leukocyte chemotaxis induction, carrying out a pro-inflammatory activity, and hematopoietic stem cell proliferation inhibition (74). The heatmap in the different groups analyzed (**Figure 1**) revealed the presence of two distinct subgroups within the group NR-NT, one of which is characterized by high cytokine and chemokine concentrations, and consists exclusively of NSCLC patients. On the other hand, UMs are exclusively present in groups without cumulative toxicity irrespective of the responding status. UMs constitute a peculiar pathological entity with respect to cutaneous melanomas, with specific immune regulatory mechanisms yet to be fully understood.

Overall, a specific signature in terms of network connectivity characterized by significant and specific connections was highlighted. In particular, it is of interest to note that the highest number of both significant connections (143) and specific connections (80) were recorded in the NR-T patients group. Furthermore, it is the only group in which IDO is involved in 4 specific and significant connections; conversely IDO is absent in the specific connections of the other 3 networks, confirming its central role in immunosuppression, tumor immune escape and in the development of primary resistance to treatment with ICIs (75).

In this study, based on a heterogeneous population in terms of primary tumor and prognosis, previous treatments, lines of treatment (first or second line), a pattern of dysregulation of the immune system, was evidenced. The immune status of patients, although significantly conditioned by organ-specific immunity as well as by previous treatments and by the lines of treatment, has a signature characterized by specific connections between the analyzed molecules. This specific immune-signature characterized non responder patients and patients prone to develop immune-related toxicity. The size of the patient sample and the non-homogeneous distribution of the different tumor types represented the main limitation of this study. Therefore, this finding should be confirmed on a larger and more homogeneous population.

The possibility of early identification of patients resistant to immunotherapy and at risk of developing high grade toxicities, would have a significant impact on clinical practice, which deserves further investigations.

## 5 Conclusion

A specific connectivity pattern for each of the clinical groups analyzed was found. In particular, in patients who will develop irAEs, a peculiar pattern of immune dysregulation was identified. The analysis of the connectivity network has shown that patients with the worst prognosis (NR-T) have peculiar connectivity and network characteristics, which could favor further research for their early identification to modulate the therapeutic strategy based on the patient immune status. A poorly modulated and highly “inflamed” immune system could ultimately affect both immune tolerance with the onset of irAE and resistance to treatment.This study suggests for the first time the possible predictability of tumor response and irAEs onset based on a specific signature, shaped by the interconnections between circulating immune system regulatory molecules.The identification of a baseline soluble immune profile, predicting both the risk of developing immune-related toxicities and worse response to treatment, represents a new challenge for precision medicine in order to design a customized therapeutic strategy, in order to prevent life-threatening irAEs and improve outcomes.

## Data availability statement

The raw data supporting the conclusions of this article will be made available by the authors, without undue reservation.

## Ethics statement

The studies involving human participants were reviewed and approved by local ethics committee of Sapienza University of Rome. The patients/participants provided their written informed consent to participate in this study.

## Author contributions

SM and GP equally contributed to the work. Conceptualization, SM and GP. methodology, AB, SM and GP. formal analysis, GF. investigation, MN, AR, CN, IZ. resources: MN. data curation, GP, ER, GS, GT, SA, IZ, CN, AR. writing—original draft preparation, GP, SM and GF. writing—review and editing, DS, SA, GD, ER, GT, GS, GL. visualization, GF and GP. supervision, AB, SM, GL, DS and GD. All authors have read and agreed to the published version of the manuscript. All authors contributed to the article and approved the submitted version.

## Supplementary material

Supplementary Table 1 xlsx This table is composed of 4 sheets reporting all the connectivity networks for responder patients at T0 as edge-lists, along with the correlation values and corresponding p-values. In particular, the first sheet lists the molecules’ network connections for responder patients without toxicity (depicted in **Figure 5A**); the second sheet lists the molecules’ network connections for responder patients with toxicity (depicted in **Figure 5B**).

Supplementary Table 2 xlsx This table is composed of 3 sheets reporting all the connectivity networks for not responder patients at T0 as edge-lists, along with the correlation values and corresponding p-values. In particular, the first sheet lists the molecules’ network connections for non-responder patients without toxicity (depicted in **Figure 5C**); the second sheet lists the molecules’ network connections for non-responder patients with toxicity (depicted in **Figure 5D**).

Supplementary Table 3 xlsx This table is composed of 5 sheets, the first one including the molecules’ network connections shared among the four patient groups (i.e., NR-T, NR-NT, R-T, T-NT), the other ones including the network connections specifically present in each group of patients.
